# Dialogic Book-Sharing as a Privileged Intersubjective Space

**DOI:** 10.3389/fpsyg.2022.786991

**Published:** 2022-03-03

**Authors:** Lynne Murray, Holly Rayson, Pier-Francesco Ferrari, Sam V. Wass, Peter J. Cooper

**Affiliations:** ^1^School of Psychology and Clinical Language Sciences, University of Reading, Reading, United Kingdom; ^2^Institute des Sciences Cognitives Marc Jeannerod (CNRS), Bron, France; ^3^Dipartimento di Neuroscienza, Universitá di Parma, Parma, Italy; ^4^School of Psychology, University of East London, London, United Kingdom

**Keywords:** intersubjectivity, dialogic book-sharing, infant attention, joint attention, language learning, pointing, gaze, parent-infant interaction

## Abstract

Parental reading to young children is well-established as being positively associated with child cognitive development, particularly their language development. Research indicates that a particular, “intersubjective,” form of using books with children, “Dialogic Book-sharing” (DBS), is especially beneficial to infants and pre-school aged children, particularly when using picture books. The work on DBS to date has paid little attention to the theoretical and empirical underpinnings of the approach. Here, we address the question of what processes taking place during DBS confer benefits to child development, and why these processes are beneficial. In a novel integration of evidence, ranging from non-human primate communication through iconic gestures and pointing, archaeological data on Pre-hominid and early human art, to experimental and naturalistic studies of infant attention, cognitive processing, and language, we argue that DBS entails core characteristics that make it a privileged intersubjective space for the promotion of child cognitive and language development. This analysis, together with the findings of DBS intervention studies, provides a powerful intellectual basis for the wide-scale promotion of DBS, especially in disadvantaged populations.

## Introduction

Disparities in children's literacy and educational achievements are of global public concern (Walker et al., 2011; Garcia and Weiss, 2017). Their roots are evident early in development, with substantial differences in language skills associated with family socio-economic status and parent education apparent by just 24 months (Fernald et al., 2013; Justice et al., 2020). Such early differences in infant cognitive functioning persist (Bornstein, 2014) and influence the life trajectory, including future education and employment (Fagan et al., 2007).

Educational disadvantage is transmitted across generations, with poor outcomes largely explained by aspects of the home environment (Sylva, 2014; Sammons et al., 2015). One important aspect is early parental reading to the child (e.g., Bus et al., 1995; Demir-Lira et al., 2019; Leech et al., 2022), a practice that varies widely between families (Logan et al., 2019). Indeed, a notable U.S. intergenerational longitudinal study showed that the strong association between parents' education achievement and that of their offspring when aged 29 years was accounted for by how much the parents had read to their child before they started school (Gottfried et al., 2015). Given such evidence, there have been efforts to promote parental reading by providing families with books from birth through the early years [e.g., Reach out and Read (Zuckerman, 2009), Book-start (https://bookstart.org.uk), Book Dash (www.bookdash.org), Mikhulu Trust (www.mikhulutrust.org)].

Aside from the simple amount of book-reading parents do with their children, *how* books are used is important. In particular, “dialogic” reading, or dialogic book-sharing (DBS), appears especially advantageous to children's language and literacy skills (e.g., Whitehurst et al., 1988; Bus et al., 1995; Hargrave and Sénéchal, 2000). This practice, which is very different from simply reading a book to a child who passively listens (Peskin and Astington, 2004), also varies across families, being less common in those that are more disadvantaged (Bus et al., 1995; Fletcher and Reese, 2005).

## The Intersubjective Characteristics of DBS

**Figure 1:**
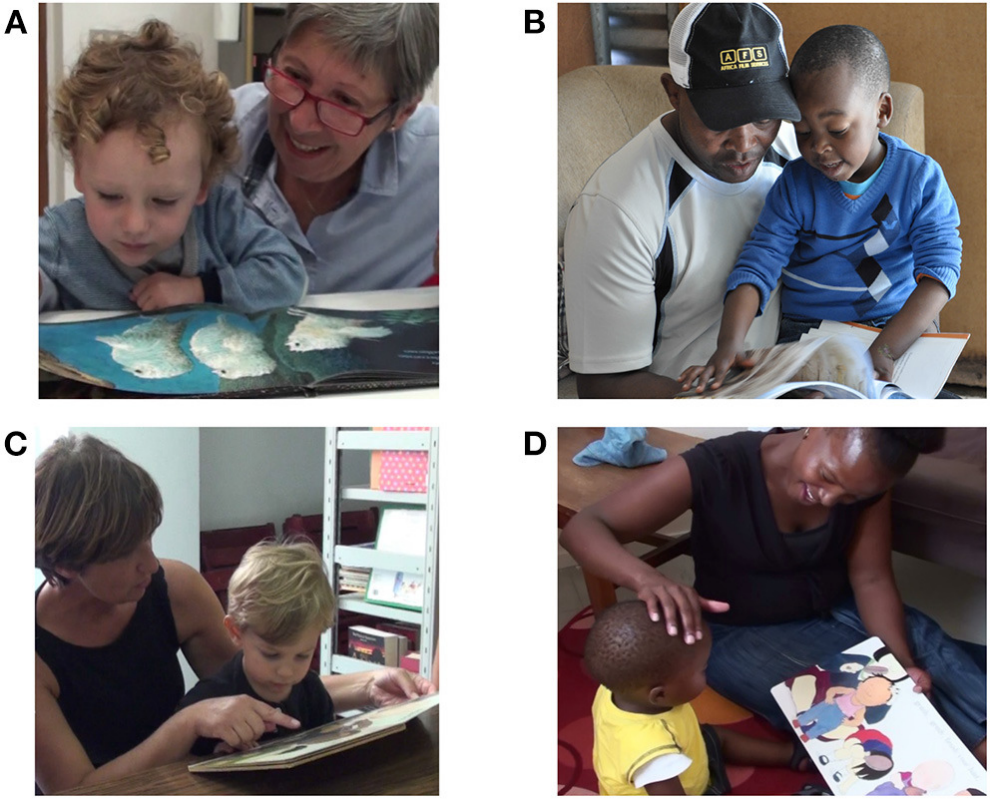
Typical affectionate intersubjective behaviors in Dialogic Book-sharing, illustrated from the authors' training materials: **(A)** Following child gaze; **(B)** Following child pointing; **(C)** Pointing to focus of child interest and naming-elaborating; **(D)** Linking book-content to child experience and animating.

Young children's learning is fundamentally dyadic (Vygotsky, 1978). From the first weeks, infants engage in rich “primary intersubjective” face-to-face communication with their carers (Trevarthen, 1979), followed by a “secondary intersubjective” phase around 9-10 months characterized by shared attention to common referents (Trevarthen and Hubley, 1978; Tronick et al., 1979; Abney et al., 2020). Sharing picture-books typically starts in this latter phase, and is an intersubjective process in which books are used to support the child's interest and engage them in a reciprocal interaction. Book-sharing provides a contained space for joint attention in a physically close intimate setting that is associated with the secure attachment (Bus and van IJzendoorn, 1995, 1997) and shared physiological and affectively positive states (Waters et al., 2017) that promote cognitive and language development (Van IJzendoorn et al., 1995). Core characteristics of DBS are that the adult pays attention to what the child is interested in, follows their interest, and builds upon this in an emotionally supportive way that actively involves the child. Aside from gazing at and pointing to what the child is looking at and naming it, adult DBS behaviors include asking questions and pitching comments according to the child's developmental capacity (Vygotsky, 1978), linking the book content to the child's own experience, and supporting their interest through use of animated vocalizations and gestural enactment (Whitehurst et al., 1988; Cooper et al., 2014; Vally et al., 2015; see Figure 1).

Given the benefits of DBS, a number of programmes for training carers in this method have been developed. A recent meta-analysis of 19 randomized controlled trials of DBS training, including in highly disadvantaged communities, reported a large effect on caregiver book-sharing quality (mean *d* = 1.01); and, regarding child outcomes, it showed benefits to both expressive and receptive language, across the age range (12–60 months) (mean *d* = 0.41 and 0.26, respectively) (Dowdall et al., 2020). There is also evidence for a benefit of training parents in DBS on infant focal attention (Cooper et al., 2014; Vally et al., 2015), an important component of general cognitive processing (Smith, 2013) and a key predictor of scholastic functioning (McClelland et al., 2013). Importantly, intervention studies have shown that it is by virtue of the improvements in parent-infant book-sharing interactions effected by training that the benefits to child language and attention are brought about (Murray et al., 2016).

Here, we present speculation and evidence from independent research that may explain why DBS, particularly when using text-free or text-light picture books, is such an effective, or privileged, mode of supporting early development. First, we consider the possible evolutionary precursors and ontogenetic development of the capacity to harness picture-book images in the service of sharing meanings with others. We then focus on specific intersubjective, joint attention-relevant behaviors that are prominent in DBS, and consider their role in the development of child cognitive functioning. Finally, we note the linguistic characteristics of DBS.

## The Privileged Nature of Book-Sharing

### Evolutionary and Developmental Precursors of Iconic Understanding

The development of shared understanding through iconic forms appears to have a long evolutionary history, and this could be a powerful driver of the capacity of infants and young children to apprehend the spatial arrangement of marks on a surface, as in picture books, to share reference to objects, individuals, or events in the real world. Thus, studies in the wild of the gestures used by non-human primates show that, despite the potential for numerous hand and limb configurations, there is a common repertoire of gestures across widely dispersed species and populations, each one carrying similar meaning (Corballis, 2010; Hobaiter et al., 2014; Byrne et al., 2017; Graham et al., 2018). Notably, these gestures are characterized by their “iconic” spatial configuration as the limb is moved through space. Research also shows apes can be trained to use iconic symbols, deploying them flexibly in exchanges with humans (Bohn et al., 2016). With regard to early hominid use of iconic communication, deliberate markings on surfaces were used even by pre-Homo sapiens, as shown in Neanderthal cave art in the form of a hand stencil (minimum age 66.7 ka) and a scalariform sign (minimum age 64.8 ka) in Maltravieso (Hoffmann et al., 2018). In early Homo sapiens history, 43.9 ka, in Indonesia, in what is the oldest-known parietal art by modern humans, pictorial “narratives” depict what seems to be a communal hunt, with human-like figures using spears and/or ropes to flush animals from their cover toward waiting hunters (Aubert et al., 2019). This scene, regarded as the earliest evidence of communication of a narrative in Paleolithic art, is particularly notable because the invention of fictional stories may have been the last and most critical stage in the evolutionary history of human language and the development of modern cognition (Mithen, 2009; Boyd, 2018; Aubert et al., 2019).

### Ontological Development of the Apprehension of Iconic Forms

**Figure 2:**
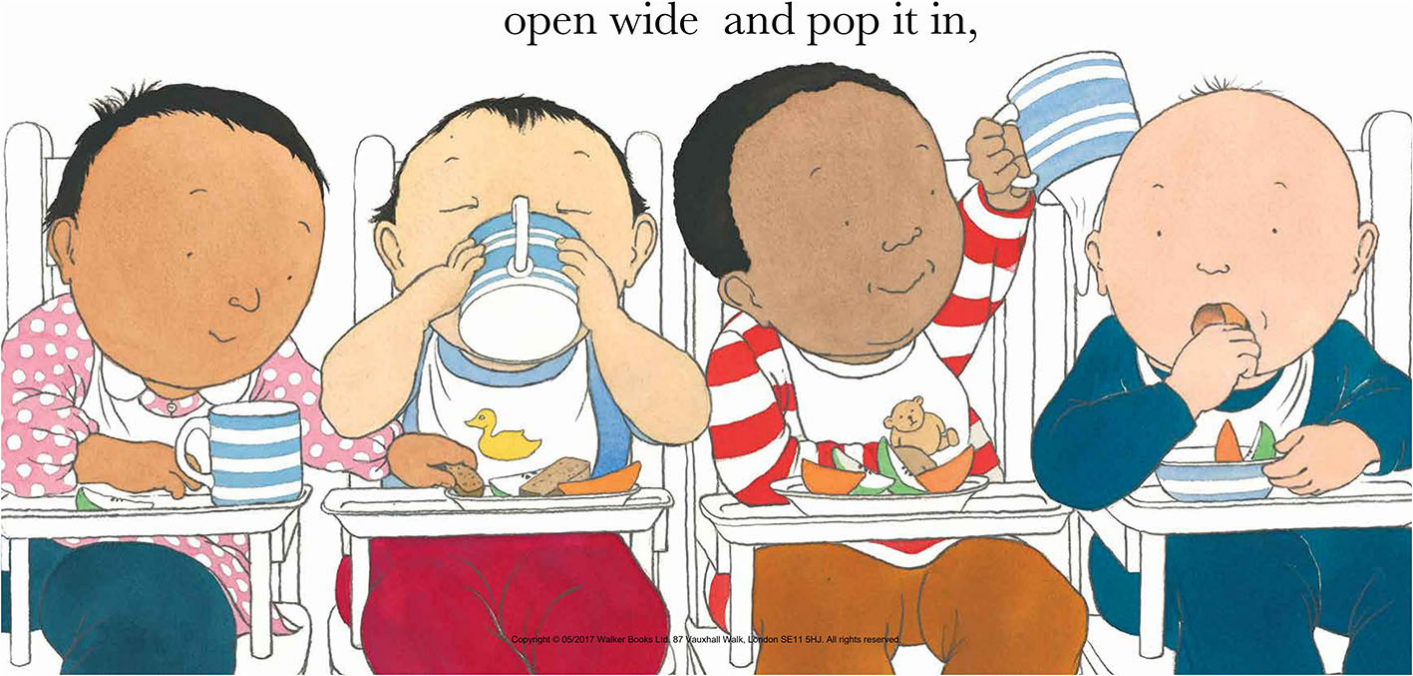
This beautiful illustration by Oxenbury (1987) provides clear, repeated prototypical depictions of familiar categories of subjects, objects and actions whose reference can be readily apprehended by infants from around 1 year. It also presents parents with excellent opportunities for different levels of verbal scaffolding, according to their infant's age and experience. These can range from providing simple labels for the youngest infants (“baby,” “cup,” “drinking”), to linking to the infant's own experience and elaborating (look, that baby's got a cup like yours'), to talking about perspectives and mental states for older infants and children (Does this baby know that his biscuit's being taken? How does that baby feel when the other baby pours milk on his head?!). Copyright © 1987 Helen Oxenbury From CLAP HANDS by Helen Oxenbury Reproduced by permission of Walker Books Ltd, London, SE11 5HJ www.walker.co.uk.

The strikingly rapid ontological development of the ability to connect 2D images to their referents, possibly drawing on the pre- and early human evolution of iconic communication, has been well-charted in experimental research. For example, infants can recognize their mother's photograph by just 3 months (Barrera and Maurer, 1981), can use a picture to identify a specific object by 15 months (Preissler and Bloom, 2008; Ganea et al., 2009) and a generic object by 15–17 months (Geraghty et al., 2014); and by 18–24 months they can use just sparse visual information to recognize well-known objects (Smith, 2009). Remarkably, by the same age, infants can use a verbal label previously paired with a line drawing of an unknown object to select the referent object, in preference, even, to the familiar line drawing itself (Preissler and Carey, 2004). Finally, by 3 years, children are able to accept abstract line drawings as reflecting the drawer's intended referent, even when the drawing shows little, if any, physical resemblance to the object (Smith, 2003, 2013; Hartley and Allen, 2014). Concerning the apprehension of actions, although infants can infer intentionality from observation of abstract symbols in motion (Biro et al., 2007; Pomiechowska and Csibra, 2017), evidence is lacking concerning *static* arrays. Nevertheless, studies of adults indicate that only minimal two dimensional marks on a surface are required to detect intentionality, perhaps supported by a neural action observation network (AON) (Umiltà et al., 2012). Such activity may reflect a motor simulation mechanism, whereby the observation of deliberate marks produced by another person produces a first-person embodied experience. The fact that the relevant AON appears operational in infancy for manual gestures and facial expressions (Rayson et al., 2017; Debnath et al., 2019), make it plausible that the ability to apprehend intentionality, and possibly other mental states (e.g., basic emotions), from two dimensional depictions in picture books is in place by late infancy. This is particularly likely where the picture content is well-organized, and uses prototypical cues to depict the various categories of familiar objects, actions and emotions (see Figure 2).

### Joint Attention in Book-Sharing

Joint attention in general is associated with a wide range of positive effects, both cognitive (Shteynberg, 2018) and emotional (Schilbach et al., 2010; Wolf et al., 2016). Establishing and maintaining a state of joint attention between adult and child is fundamental to good book-sharing, and below we consider specific aspects of joint attention that are prominent in this context.

#### Gaze Following

As noted, a key feature of DBS is the parent following their infant or young child's direction of interest. Experimental research across the age range has demonstrated the beneficial effects of having one's gaze followed for core aspects of emotional and cognitive functioning. Thus, evidence suggests that there is an expectation that our own gaze will be followed, with distinct motivational consequences when this occurs. Awareness that our gaze has been followed takes place very quickly, in less than half a second (Phillips et al., 2022), and even this brief time is experienced as compressed, via a “temporal, or intentional, binding effect” (David et al., 2008). Relatedly, when another person's gaze-shift occurs rapidly after our own, we sense it as being *connected* to our own gaze, and this leads to a positive, implicit sense of our agency (Pfeiffer et al., 2012; Haggard, 2017; Stephenson et al., 2018). Cognitive processing of the target of our gaze is also improved when our gaze to it is followed, vs. following another person's gaze, an effect that is evident from early infancy. For example, when a 6.5-month-old infant's own gaze to an object is followed, vs. being cued, by another's gaze, they show enhanced neural processing, as reflected in increased EEG-recorded alpha mu suppression (Rayson et al., 2019); and, under the same conditions, 10–12-month-olds show behavioral indices of efficiency of information processing (gaze-shift speed) and object preference (Ishikawa et al., 2019).

Consistent with experimental findings, naturalistic studies show enhanced infant attention and neural processing (alpha suppression) of objects during joint play, vs. solo play or simple observation (Wass S. et al., 2018; Meyer et al., 2022). Moreover, during joint play, when the parent visually attends to the infant's object of interest, infants extend the duration of their visual attention to the object, particularly if the parent's interest is sustained (Yu and Smith, 2016). Such effects seem to be mediated by the *nature* of the adult's attention to the infant: dual EEG measurement with 12-month-old infants and their parents showed that parents' theta power closely tracked and responded to changes in their infants' gaze direction, and instances where parents showed increased neural responsivity were associated with longer periods of infant sustained attention (Wass S. V. et al., 2018).

#### Pointing

Around 9–12 months, infants come to understand pointing as object-directed, and their own pointing is related to their understanding of this property of others' points (Woodward and Guajardo, 2002). Developing this capacity might profit particularly well from the book-sharing context with its potential for sustained periods of joint attention to a series of targets. Indeed, pointing to the elements of picture book displays is a common feature of book-sharing behavior on the part of both infants and parents. Recent experimental studies have shown that, as for gaze following, significant benefits accrue from experiences involving pointing (e.g., Salo et al., 2019). Thus, at the neural level, when a target has attracted 8-month-old infants' attention, larger amplitude P400 ERP components are observed if the target location is then cued with a point (Gredeback et al., 2010). Benefits are also apparent when it is the infant who performs the point: even “solo” pointing can help infants' attention processing (Smith, 2013), though they typically point when others are available to respond (Begus and Southgate, 2012); and having their own pointing followed is associated with subsequent gains in vocabulary (Brooks and Meltzoff, 2008) and better learning (as indexed by imitation) of others' novel object-directed actions (Begus et al., 2014). Such benefits, like those of having one's gaze followed, may, in part, accrue from “action-oriented predictive processing” effects, whereby one's motor intentions elicit predictions about the results of our actions (Clark, 2013), with the subsequent, anticipated, events then evoking increased neural responsiveness (Engel et al., 2001). These mechanisms, largely studied under experimental conditions, could potentially occur in natural social interactions (de Hamilton, 2021; Monroy C. et al., 2021), including in book-sharing, such that infant attentional and gestural behaviors entailing anticipation and prediction of parental responses then elicit greater neural activation when those responses occur (Southgate et al., 2009; Monroy C. D. et al., 2019; Phillips et al., 2022).

#### Naming and Animating

Although an adult simply pointing to a target can influence infant attention (Butterworth, 2004), its effects when used in isolation from other behaviors may be limited. In fact, parental pointing during spontaneous parent-infant interactions is often part of a more complex display, including during book-sharing. Indeed, pointing combined with “naming” occurs more commonly in book-sharing than in any other conversational context (Dunn and Wooding, 1977), and is regarded as key to book-sharing's function as a “language acquisition device” (Ninio and Bruner, 1978; Ninio, 1983). Although basic associative processes may contribute to the word-learning afforded by pointing plus naming, the occurrence of this behavior during book-sharing is typically more dynamic than a simple temporal coincidence of auditory and deictic stimuli (Meyer et al., 2011). Thus, parents often use intonational and facial modulation for emphasis as they name the target of their pointing (Nencheva et al., 2021), as well as synchronized gestural animation (Novack and Goldin-Meadow, 2017), particularly when naming depicted actions. As such, the book-sharing context typically provides infants with highly enriched inter-sensory information.

Studies of infant attention to actual as opposed to depicted objects, and of word-object learning, confirm the value of the sort of “inter-sensory redundancy” (Gogate et al., 2001) that occurs in book-sharing. For example, within the first year, effects on infant attention of having objects pointed to are enhanced by the addition of vocal communication (Daum et al., 2013), and better learning of object-sounds/proto-word associations occurs when their presentations are synchronized (Gogate and Bahrick, 1998), or the object dynamics suggest animation or deixis (e.g., “looming”; Matatyaho-Bullaro et al., 2014). Further, if caregivers name objects with synchronous movement, vs. asynchronous or no movement, infants are more likely to attend to the object, look between object and parent, and show better word learning (Gogate et al., 2006).

Aside from this human infancy research, the potential benefits of “embodied,” or gesturally enacted communication for spoken language acquisition are also suggested by work on non-human primate communication and sign language. First, the *iconic* properties of primate and human-signed gestures suggest a more direct relation to referential spoken language than do non-referential vocalizations (Corballis, 2010; Perniss and Vigliocco, 2014). Second, neurological research shows that AON regions implicated in hand and arm movements are closely located to those for mouth movements, suggesting the possibility of a close functional relationship (Fogassi and Ferrari, 2007; Corballis, 2010).

### Special Linguistic Characteristics of Book-Sharing

In addition to “pointing and naming,” it is well-established that certain forms of parental speech are privileged in the book-sharing context (e.g., Hoff-Ginsberg, 1991; Adrian et al., 2005; Salo et al., 2016; Noble et al., 2018). This is particularly so when using picture books where, rather than relying on a prespecified text, parents instead construct their own account of the book content and adjust it to their child (Sénéchal et al., 1995) in a process of “meaning-making” (Tronick, 2009). Importantly, these speech characteristics are precisely the ones that best promote child language development [being responsive to the infant's behavior and vocalizations, elaborative, and soliciting of child involvement (Snow and Ferguson, 1977)], and that foster child socio-cognitive understanding [mental state terms, complement clauses that include the content of someone's thoughts (Peskin and Astington, 2004; Brandt et al., 2016; Devine and Hughes, 2019; Boeg Thomsen et al., 2021)]. These speech forms in DBS are embedded in dynamic intersubjective exchanges with the child, in tandem with animated vocalizations and gestures, as described above; and they help scaffold the infant's attention and their understanding of the book content by highlighting individual elements of the picture and relating them to each other in a way that is constantly adjusted to the child's age, competence and wider experience, as well as their concurrent behavior (see Figure 2).

## Discussion

A natural propensity to share meaning via iconic forms developed in our early evolutionary history, advancing new kinds of cognition and communication, including protolanguage. We argue that this natural propensity can be harnessed, even in young infants, by the provision of books with pictorial content, and powerfully exploited to enhance infants' wider cognitive development via DBS, with specific adult behaviors (e.g., gaze-following) having been shown to benefit foundational skills for child literacy and educational progress. While some of this evidence derives from experimental studies investigating single aspects of adult behavior, DBS is an intersubjective process of dynamic engagement, with each partner adjusting what they do to the other's interest and emotional expressions and, in the case of parents, their child's competence and wider experience. Notably, while constituting “intuitive parenting,” adult DBS practices can remain latent unless facilitated by support for parents' awareness of infant experience and capacities, and by guidance in the use of specific techniques. This is particularly likely where intuitive parenting practices are strained, for example, by adversity or mental health problems, or where local cultures prioritize different parenting and developmental goals (Murray et al., 2019). Accordingly, it is important that effective training programmes have been developed that promote good DBS practice and improved child outcome (Dowdall et al., 2020), particularly in contexts where low literacy rates and educational failure are major problems. While our discussion has mainly concerned evidence from WEIRD (Henrich et al., 2010) populations, and more investigation is required from wider cultural contexts to identify other patterns of parent-child interaction that are also developmentally beneficial (see Akhtar and Gernsbacher, 2008), it is nevertheless the case that, in the current global climate, literacy is the single most powerful route out of poverty (UNESCO Institute for Statistics (UIS) the Global Education Monitoring (GEM) Report, 2017; Dowd et al., 2018; RISE, 2020), particularly for girls, and opening up this potential to disadvantaged populations—for example via promotion of book-sharing—stands to be a powerful way to reduce economic inequality.

## Ethics Statement

Written informed consent was obtained from the individual(s), and minor(s)' legal guardian/next of kin, for the publication of any potentially identifiable images or data included in this article.

## Author Contributions

LM produced the first draft of the manuscript. All other authors contributed equally to developing the line of argument and the editing to produce the final manuscript.

## Conflict of Interest

The authors declare that the research was conducted in the absence of any commercial or financial relationships that could be construed as a potential conflict of interest.

## Publisher's Note

All claims expressed in this article are solely those of the authors and do not necessarily represent those of their affiliated organizations, or those of the publisher, the editors and the reviewers. Any product that may be evaluated in this article, or claim that may be made by its manufacturer, is not guaranteed or endorsed by the publisher.

## References

[B1] Abney D. H. Suanda S. H. Smith L. B. Yu C. (2020). What are the building blocks of parent–infant coordinated attention in free-flowing interaction?. Infancy 25, 871–887. 10.1111/infa.12365 33022842 PMC7934178

[B2] Adrian J. Clemente R. Villanueva L. Rieffe C. (2005). Parent-child picture book reading, mothers' mental state language and children's theory of mind. J. Child Lang. 32, 673–686. 10.1017/S0305000905006963 16220639

[B3] Akhtar N. Gernsbacher M. A. (2008). On privileging the role of gaze in infant social cognition. Child Dev. Pers. 2, 59–65. 10.1111/j.1750-8606.2008.00044.x 25520748 PMC4266544

[B4] Aubert M. Lebe R. Oktaviana A. A. Tang M. Burhan B. Hamrullah. (2019). Earliest hunting scene in prehistoric art. Nature 576, 442–445. 10.1038/s41586-019-1806-y 31827284

[B5] Barrera M. E. Maurer D. (1981). Recognition of mother's photographed face by the three-month-old infant. Child Dev. 52, 714–716. 10.2307/1129196

[B6] Begus K. Gliga T. Southgate V. (2014). Infants learn what they want to learn: responding to infant pointing leads to superior learning. PLoS ONE 9,e108817. 10.1371/journal.pone.0108817 25290444 PMC4188542

[B7] Begus K. Southgate V. (2012). Infant pointing serves an interrogative function. Dev. Sci. 15, 611–617. 10.1111/j.1467-7687.2012.01160.x 22925509

[B8] Biro S. Csibra G. Gergely G. (2007). The role of behavioral cues in understanding goal-directed actions in infancy. Prog. Brain Res. 164, 303–322. 10.1016/S0079-6123(07)64017-5 17920439

[B9] Boeg Thomsen D. Theakston A. Kandemirci B. Brandt S. (2021). Do complement clauses really support false-belief reasoning? A longitudinal study with English-speaking 2- to 3-year-olds. Dev. Psychol. 57, 1210–1227. 10.1037/dev0001012 34591566 PMC9330672

[B10] Bohn M. Call J. Tomasello M. (2016). Comprehension of iconic gestures by chimpanzees and human children J. Exp. Child Psychol. 142, 1–17. 10.1016/j.jecp.2015.09.001 26448391

[B11] Bornstein M. H. (2014). Human infancy…. And the rest of the lifespan. Ann. Rev. Psychol. 65, 121–158. 10.1146/annurev-psych-120710-100359 24405360 PMC5865600

[B12] Boyd B. (2018). The evolution of stories: from mimesis to language, from fact to fiction. Wiley Interdiscip. Rev. Cogn. Sci. 9, e1444. 10.1002/wcs.1444 28544658 PMC5763351

[B13] Brandt S. Buttelmann D. Lieven E. Tomasello M. (2016). Children's understanding of first- and third-person perspectives in complement clauses and false-belief tasks. J. Exp. Child Psychol. 151, 131–143. 10.1016/j.jecp.2016.03.004 27067632

[B14] Brooks R. Meltzoff A. N. (2008). Infant gaze following and pointing predict accelerated vocabulary growth through two years of age: a longitudinal, growth curve modelling study. J. Child Lang. 35, 207–220. 10.1017/S030500090700829X 18300435

[B15] Bus A. G. van IJzendoorn M. H. (1995). Mothers reading to their three-year-olds: the role of mother-child attachment security in becoming literate. Read. Res. Q. 30, 998–1015.

[B16] Bus A. G. van IJzendoorn M. H. (1997). Affective dimensions of mother-infant picture book reading. J. Sch. Psychol. 35, 47–60.

[B17] Bus A. G. van IJzendoorn M. H. Pellegrini A. (1995). Joint book-reading makes for success in learning to read: a meta analysis on intergenerational transmission of literacy. Rev. Educ. Res. 65, 1–21.

[B18] Butterworth G. (2004). Theories of Infant Development. eds G. Bremner and A. Slater. Oxford: Blackwell Publications, 317–354.

[B19] Byrne R. W. Cartmill E. Genty E. Graham K. E. Hobaiter C. Tanner J. (2017). Great ape gestures: intentional communication with a rich set of innate signals. Anim. Cogn. 20, 755–769. 10.1007/s10071-017-1096-4 28502063 PMC5486474

[B20] Clark A. (2013). Whatever next? Predictive brains, situated agents, and the future of cognitive science. Behav. Brain Sci. 36, 181–204. 10.1017/S0140525X12000477 23663408

[B21] Cooper P. J. Vally Z. Cooper H. Radford T. Sharples A. Tomlinson M.. (2014). Promoting mother–infant book sharing and infant attention and language development in an impoverished South African population: a pilot study. Early Child Educ. J. 42, 143–152. 10.1007/s10643-013-0591-8

[B22] Corballis M. C. (2010). The gestural origins of language. Cogn. Sci. 1, 2–7. 10.1007/978-4-431-79102-7_2 26272832

[B23] Daum M. M. Ulber J. Gredeback G. (2013). The development of pointing perception in infancy: effects of communicative signals on covert shifts of attention. Dev. Psychol. 49, 1898–1908. 10.1037/a0031111 23356522

[B24] David N. Newen A. Vogeley K. (2008). The “sense of agency” and its underlying cognitive and neural mechanisms. Conscious. Cogn.17, 523–534. 10.1016/j.concog.2008.03.004 18424080

[B25] de Hamilton A. F. (2021). Hyperscanning: beyond the hype. Neuron 109, 404–407. 10.1016/j.neuron.2020.11.008 33259804

[B26] Debnath R. Salo V. C. Buzzell G. A. Yoo K. H. Fox N. A. (2019). Mu rhythm desynchronization is specific to action execution and observation: evidence from time-frequency and connectivity analysis. Neuroimage 184, 496–507. 10.1016/j.neuroimage.2018.09.053 30248457 PMC6261430

[B27] Demir-Lira Ö. E. Applebaum L. R. Goldin-Meadow S. Levine S. C. (2019). Parents' early book reading to children: relation to children's later language and literacy outcomes controlling for other parent language input. Dev.Sci. 22, e1274. 10.1111/desc.12764 30325107 PMC6927670

[B28] Devine R. T. Hughes C. (2019). Let's talk: parents' mental talk (not mind-mindedness or mindreading capacity) predicts children's false belief understanding. Child Dev. 90, 1236–1253. 10.1111/cdev.12990 29115674

[B29] Dowd A. J. Pisani L. Dusabe C. Howell H.-J. (2018). UNICEF: EducationThinkPieces_3_Parents-and-caregivers.pdf(unicef.org).

[B30] Dowdall N. Melendez-Torres G. J. L. Murray L. Gardner F. Hartford L. Cooper P. J. (2020). Shared picture book reading interventions for child language development: a systematic review and meta-analysis. Child Dev. 91, 383–399. 10.1111/cdev.13225 30737957

[B31] Dunn J. Wooding C. (1977). Play in the Home and Its Importance for Learning in Biology of Play. eds B. Tizard and D Harvey. Philadelphia, PA: Lippincott.

[B32] Engel A. K. Fries P. Singer W. (2001). Dynamic predictions: oscillations and synchrony in top–down processing. Nat. Rev. Neurosci. 2, 704–716. 10.1038/35094565 11584308

[B33] Fagan J. Holland C. Wheeler K. (2007). The prediction from infancy of adult IQ and achievement. Intelligence 35, 225–231. 10.1016/j.intell.2006.07.0078783062

[B34] Fernald A. Marchman V. A. Weisleder. (2013). A SES differences in language processing skill and vocabulary are evident at 18 months. Dev. Sci. 16, 234–248. 10.1111/desc.12019 23432833 PMC3582035

[B35] Fletcher K. L. Reese E. (2005). Picture book reading with young children: a conceptual framework. Dev. Rev. 25, 64–103. 10.1016/j.dr.2004.08.009

[B36] Fogassi L. Ferrari P. F. (2007). Mirror neurons and the evolution of embodied language. Curr. Dir. Psychol. Sci. 16, 136–141. 10.1111/j.1467-8721.2007.00491.x

[B37] Ganea P. A. Allen M. L. Butler L. Carey S. DeLoache J. S. (2009). Toddlers' referential understanding of pictures. J. Exp. Child Psychol. 104, 283–295. 10.1016/j.jecp.2009.05.008 19560783 PMC2865246

[B38] Garcia E. Weiss E. (2017). Reducing and Averting Achievement Gaps. Key Findings From the Report ‘Education Inequalities at the School Starting Gate’ and Comprehensive Strategies to Mitigate Early Skills Gaps. London: Economic Policy Institute.

[B39] Geraghty K. Waxman S. R. Gelman S. A. (2014). Learning words from pictures: 15- and 17-month-old infants appreciate the referential and symbolic links among words, pictures, and objects. Cogn. Dev. 32, 1–11. 10.1016/j.cogdev.2014.04.003

[B40] Gogate L. J. Bahrick L. E. (1998). Intersensory redundancy facilitates learning of arbitrary relations between vowel sounds and objects in seven-month-olds. J. Exp. Clin. Child Psychol., 69, 133–149. 10.1006/jecp.1998.2438 9637756

[B41] Gogate L. J. Bolzani L. H. Betancourt E. A. (2006). Attention to maternal multi-modal naming by 6- to 8-month-old infants and learning of word-object relations. Infancy 9, 259–288. 10.1207/s15327078in0903_1 33412680

[B42] Gogate L. J. Walker-Andrews A. Bahrick L. E. (2001). The intersensory origins of word comprehension: an ecological-dynamic systems view. Dev. Sci. 4, 1–37. 10.1111/1467-7687.00143

[B43] Gottfried A. W. Schlackman J. Gottfried A. E. Boutin-Martinez A. S. (2015). Parental provision of early literacy environment as related to reading and educational outcomes across the academic lifespan. Parent. Sci. Pract. 15, 24–38. 10.1080/15295192.2015.992736

[B44] Graham K. E. Hobaiter C. Ounsley J. Furuichi T. Byrne R. W. (2018). Bonobo and chimpanzee gestures overlap extensively in meaning. PLoS Biol. 16, e2004825. 10.1371/journal.pbio.2004825 29485994 PMC5828348

[B45] Gredeback G. Melinder A. Daum M. (2010). The development and neural basis of pointing comprehension. Soc. Neurosci. 5, 2010. 10.1080/17470910903523327 20162491

[B46] Haggard P. (2017). Sense of agency in the human brain. Nat. Rev. Neurosci. 18, 196–207 10.1038/nrn.2017.14 28251993

[B47] Hargrave M. Sénéchal A. C. (2000). A book reading intervention with preschool children who have limited vocabularies: the benefits of regular reading and dialogic reading. Early Child Res. Q. 15, 75–90. 10.1016/S0885-2006(99)00038-1

[B48] Hartley C. Allen M. L. (2014). Intentions vs. resemblance: understanding pictures in typical development and autism. Cognition 131, 44–59. 10.1016/j.cognition.2013.12.009 24440433

[B49] Henrich J. Heine S. Norenzayan A. (2010). The weirdest people in the world? Behav. Brain Sci. 33, 61–83. 10.1017/S0140525X0999152X 20550733

[B50] Hobaiter C. Leavens D. A. Byrne R. W. (2014). Deictic gesturing in wild chimpanzees (*Pan troglodytes*)? Some possible cases. J. Comp. Psychol. 128, 82–87. 10.1037/a0033757 24040760

[B51] Hoff-Ginsberg E. H. (1991). Mother-child conversation in different social classes and communicative settings. Child Dev. 62, 782–796. 1935343 10.1111/j.1467-8624.1991.tb01569.x

[B52] Hoffmann D. L. Standish D. Gardia-Diez M. Pettittt P. B. Milton J. A. Jilhao J.. (2018). U-Th dating of carbonate crusts reveals Neandertal origin of Iberian cave art. Science 359, 912–915. 10.1126/science.aap7778 29472483

[B53] Ishikawa M. Yoshimura M. Sato H. Itakura (2019). Effects of attentional behaviours on infant visual preferences and object choice. Cogn. Process. 20, 317–324. 30955152 10.1007/s10339-019-00918-x

[B54] Justice L. M. Jiang H. Bates R. Koury A. (2020). Language disparities related to maternal education emerge by two years in a low-income sample. Mat Chi Health J. 24, 1419–1427. 32632843 10.1007/s10995-020-02973-9 PMC7572544

[B55] Leech K. A. McNally S. Daly M. Corriveau K. H. (2022). Unique effects of book-reading at 9-months on vocabulary development at 36-months: insights from a nationally representative sample of Irish families. Early Child Res. Q. 58, 242–253. 10.1016/j.ecresq.2021.09.009

[B56] Logan J. A. R. Justice L. M. Yumuş M. Chaparro-Moreno L. J. (2019). When children are not read to at home: the million word gap. J. Dev. Behav. Ped. 40, 383–386. 10.1097/DBP.0000000000000657 30908424

[B57] Matatyaho-Bullaro D. J. Gogate L. Mason Z. Cadavid S. Abdel-Mottaleb M. (2014). Type of object motion facilitates word mapping by preverbal infants. J. Exp. Child Psychol. 118, 27–40. 10.1016/j.jecp.2013.09.010 24211772

[B58] McClelland M. Acock A. C. Piccinin A. Rhea S. A. Stallings M. C. (2013). Relations between preschool attention span-persistence and age 25 educational outcomes. Early Child Res. Q. 28, 314–324. 10.1016/j.ecresq.2012.07.008 23543916 PMC3610761

[B59] Meyer M. Chung H. Debnath R. Fox N. Woodward A. L. (2022). Social context shapes neural processing of others' actions in 9-month-old infants. J. Exp. Child Psychol. 213, 105260. 10.1016/j.jecp.2021.105260 34390926

[B60] Meyer M. Hard B. Brand R. McGarvey M. Baldwin D. (2011). Acoustic packaging: maternal speech and action synchrony. Auton. Ment. Dev. IEEE Trans. 3, 154–162. 10.1109/TAMD.2010.2103941

[B61] Mithen S. (2009). “Material culture and the supernatural,” in Becoming Human: Innovation in Prehistoric Material and Spiritual Culture, eds C. Renfrew and I. Morley (Cambridge: Cambridge Univ. Press), 123–134.

[B62] Monroy C. Chen C. Houston D. Yu C. (2021). Action prediction during real-time parent-infant interactions. Dev. Sci. 24, e13042. 10.1111/desc.13042 33030770 PMC8026764

[B63] Monroy C. D. Meyer M. Schröer L. Gerson S. A. Hunnius S. (2019). The infant motor system predicts actions based on visual statistical learning. Neuroimage 185, 947–954. 10.1016/j.neuroimage.2017.12.016 29225063

[B64] Murray L. De Pascalis L. Tomlinson M. Vally Z. Dadomo H. MacLachlan B.. (2016). Randomized controlled trial of a book-sharing intervention in a deprived South African community: effects on carer-infant interactions, and their relation to infant cognitive and socio-emotional outcome. J. Child Psychol. Psychiatr. 57, 1370–1371. 10.1111/jcpp.12605 27465028 PMC5659125

[B65] Murray L. Halligan S. L. Cooper P. J. (2019). “Postnatal depression and child development,” in Handbook of Infant Mental Health, 4th Edn, ed C Zeanah (New York, NY: NY Guilford Press), 172–186.

[B66] Nencheva M. L. Piazza E. A. Lew-Williams C. (2021). The moment-to-moment pitch dynamics of child-directed speech shape toddlers' attention and learning. Dev. Sci. 24, e12997. 10.1111/desc.12997 32441385 PMC7680269

[B67] Ninio A. (1983). Joint book reading as a multiple vocabulary acquisition device. Dev. Psychol. 19, 445–451.

[B68] Ninio A. Bruner J. (1978). The achievement and antecedents of labelling. J. Child Lang. 5, 11–15.

[B69] Noble C. Cameron-Faulkner T. Lieven E. (2018). Keeping it simple: the grammatical properties of shared book reading. J. Child Lang. 45, 753–766. 10.1017/S0305000917000447 29145915

[B70] Novack M. A. Goldin-Meadow S. (2017). Gesture as representational action: a paper about function. Psychon. Bull. Rev. 24, 652–665. 10.3758/s13423-016-1145-z 27604493 PMC5340635

[B71] Oxenbury H. (1987). Clap Hands. London: Walker Books.

[B72] Perniss P. Vigliocco G. (2014). The bridge of iconicity: from a world of experience to the experience of language. Phil. Trans. R. Soc. B. 369, 20130300. 10.1098/rstb.2013.0300 25092668 PMC4123679

[B73] Peskin J. Astington J. (2004). The effects of adding metacognitive language to story texts. Cogn. Dev. 19, 253–273. 10.1016/j.cogdev.2004.01.003

[B74] Pfeiffer U. J. Schilbach L. Jording M. Timmermans B. Bente G. Vogeley K. (2012). Eyes on the mind: investigating the influence of gaze dynamics on the perception of others in real-time social interaction. Front. Psychol. 3:537. 10.3389/fpsyg.2012.00537 23227017 PMC3512550

[B75] Phillips E. A. M. Goupil L. Marriott-Haresign I. Bruce-Gardyne E. Csolsim F. A. Whitehorn M.. (2022). Proactive or Reactive? Neural Oscillatory Insight Into the Leader-Follower Dynamics of Early Infant-Caregiver Interaction. Available online at: https://psyarxiv.com/cg38a 10.1073/pnas.2122481120 PMC10104541 37014853

[B76] Pomiechowska B. Csibra G. (2017). Motor activation during action perception depends on action interpretation. Neuropsychology 105, 84–91. 10.1016/j.neuropsychologia.2017.01.032 28189494 PMC5447367

[B77] Preissler M. A. Bloom P. (2008). Two-year-olds use artist intention to understand drawings. Cognition 106, 512–518. 10.1016/j.cognition.2007.02.002 17391660

[B78] Preissler M. A. Carey S. (2004). Do both pictures and words function as symbols for 18- and 24-month-old children? J. Cogn. Dev. 5, 185–212. 10.1207/s15327647jcd0502_2

[B79] Rayson H. Bonaiuto J. J. Ferrari P. F. Chakrabarti B. Murray L. (2019). Building blocks of joint attention: early sensitivity to having one's own gaze followed. Dev. Cogn. Neurosci. 37, p100631. 10.1016/j.dcn.2019.100631 30970289 PMC6556871

[B80] Rayson H. Bonaiuto J. J. Ferrari P. F. Murray L. (2017). Early maternal mirroring predicts infant motor system activation during facial expression observation. Sci. Rep. 7, 1–11. 10.1038/s41598-017-12097-w 28916786 PMC5601467

[B81] RISE (2020). Quality Education for Every Girl for 12 Years: Insights from RISE Programme Research. Available online at: https://riseprogramme.org/publications/quality-education-every-girl-12-years-insights-rise-programme-research

[B82] Salo V.-C. Rowe M. Leech K. A. Cabrera N. J. (2016). Low-income fathers' speech to toddlers during book reading versus toy play. J. Child Lang. 43, 1385–1399. 10.1017/S0305000915000550 26541647 PMC4860188

[B83] Salo V. C. Reeb-Sutherland B. Frenkel T. I. Bowman L. C. Rowe M. L. (2019). Does intention matter? Relations between parent pointing, infant pointing, and developing language ability. J.Cogn. Dev. 20, 635–655. 10.1080/15248372.2019.1648266 32089652 PMC7034940

[B84] Sammons P. Toth K. Sylva K. Melhuish E. Siraj I. Taggart B. L. (2015). The long-term role of the home learning environment in shaping students' academic attainment in secondary school. J. Child Serv. 10, 189–201. 10.1108/JCS-02-2015-0007

[B85] Schilbach L. Wilms M. Eickhoff S. B. Romanzetti S. Tepest R. Bente G.. (2010). Minds made for sharing: initiating joint attention recruits reward-related neurocircuitry. J. Cogn. Neurosci. 22, 2702–2715. 10.1162/jocn.2009.21401 19929761

[B86] Sénéchal M. Cornell E. H. Broda L. S. (1995). Age-related differences in the organization of parent-infant interactions during picture-book reading. Early Child Res. Q. 10, 317–337. 10.1016/0885-2006(95)90010-1

[B87] Shteynberg G. (2018). A collective perspective: shared attention and the mind. Curr. Opin. Psychol., 23, 93–97. 10.1016/j.copsyc.2017.12.007 29317182

[B88] Smith L. B. (2003). Learning to recognize objects. Psychol. Sci. 14, 244–250. 10.1111/1467-9280.03439 12741748

[B89] Smith L. B. (2009). From fragments to geometric shape: changes in visual object recognition between 18- and 24-months. Curr. Dir. Psychol. Sci. 18, 290–294. 10.1111/j.1467-8721.2009.01654.x 32489232 PMC7265591

[B90] Smith L. B. (2013). It's all connected: pathways in visual object recognition and early noun learning. Am. Psychol. 68, 1–15. 10.1037/a0034185 24320634 PMC3858855

[B91] Snow C. E. Ferguson D. (eds.). (1977). Talking to Children: Language Input and Acquisition. New York, NY: C.U.P

[B92] Southgate V. Johnson M. H. Osborne T. Csibra G. (2009). Predictive motor activation during action observation in human infants. Biol. Lett. 5, 769–772. 10.1098/rsbl.2009.0474 19675001 PMC2828001

[B93] Stephenson L. J. Edwards S. G. Howard E. E. Bayliss A. P. (2018). Eyes that bind us: Gaze leading induces an implicit sense of agency. Cognition 172, 124–133. 10.1016/j.cognition.2017.12.011 29272739

[B94] Sylva K. (2014). The role of families and pre-school in educational disadvantage. Oxf. Rev. Educ. 40, 680–695. 10.1080/03054985.2014.979581

[B95] Trevarthen C. B. (1979). “Communication and cooperation in early infancy: a description of primary intersubjectivity,” in Before Speech: the Beginning of Human Communication, ed M. Bulowa (Cambridge: Cambridge University Press), 321–347.

[B96] Trevarthen C. B. Hubley P. A. (1978). “Secondary intersubjectivity: confidence, confiding and acts of meaning in the first year”, in Action, Gesture and Symbol, ed J. Lock (London: Academic Press), 183–229.

[B97] Tronick E. (2009). “Self and dyadic expansion of consciousness, meaning-making, open systems, and the experience of pleasure,” in Coming into the World: A Dialogue Between Medical and Human Sciences, eds G. La Sala, P. Fagandini, V. Iori, and F. Monti (New York, NY; Berlin: De Gruyter), 13–24.

[B98] Tronick E. Als H. Adamson L. (1979). “Structure of early face to face communicative inter- actions,” in Before Speech: The Beginning of Interpersonal Communication, ed M. Bullowa (Cambridge: Cambridge University Press), 349–370.

[B99] Umiltà M. A. Berchio C. Sestito M Freedberg D. Gallese V. (2012). Abstract art and cortical motor activation: an EEG study. Front. Hum. Neurosci. 6:311. 10.3389/fnhum.2012.00311 23162456 PMC3499799

[B100] UNESCO Institute for Statistics (UIS) and the Global Education Monitoring (GEM) Report (2017). Reducing Global Poverty Through Universal Primary and Secondary Education. Policy paper 32 / Fact sheet 44.

[B101] Vally Z. Murray L. Tomlinson M. Cooper P. J. (2015). The impact of dialogic book-sharing training on infant language and attention: a randomized controlled trial in a deprived South African community. J. Child Psychol. Psychiat. 65, 865–873. 10.1111/jcpp.12352 25399699 PMC4552334

[B102] Van IJzendoorn M. H. Dijkstra J. Bus A. G. (1995). Attachment, intelligence, and language: a meta-analysis. Soc. Dev. 4, 115–128. 10.1111/j.1467-9507.1995.tb00055.x

[B103] Vygotsky L. (1978). Mind in Society: the Development of Higher Psychological Processes. Cambridge, MA: Harvard University Press.

[B104] Walker S. P. Wachs T. D. Grantham-McGregor S. Black M. Nelson C. A. Huffman S. L.. (2011). Inequality in early childhood: risk and protective factors for early child development. Lancet 378, 1325–1338. 21944375 10.1016/S0140-6736(11)60555-2

[B105] Wass S. Clackson S. D. Georgieva S. D. Brightman L. Nutbrown R. R. Leong R. (2018). Infants' visual sustained attention is higher during joint play than solo play: is this due to increased endogenous attention control or exogenous stimulus capture? Dev. Sci. 2, e12667. 10.1111/desc.12667 29624833

[B106] Wass S. V. Noreika V. Georgieva S. Clackson K. Brightman L. Nutbrown R.. (2018). Parental neural responsivity to infants' visual attention: how mature brains influence immature brains during social interaction. PLoS Biol. 16, e2006328. 10.1371/journal.pbio.2006328 30543622 PMC6292577

[B107] Waters S. F. West T. V. Karnilowicz H. R. Mendes W. B. (2017). Affect contagion between mothers and infants: examining valence and touch. J. Exp. Psychol. Gen. 146, 1043–1051. 10.1037/xge0000322 28493755 PMC5499532

[B108] Whitehurst G. J. Falco F. L. Lonigan C. J. Fischel J. E. DeBaryshe B. D. Valdez-Menchaca M. C.. (1988). Accelerating language development through picture book reading. Dev. Psychol. 24, 552–559.30737957

[B109] Wolf D. Launay J. Dunbar R. I. M. (2016). Joint attention, shared goals, and social bonding. Br. J. Psychol. 107, 322–337. 10.1111/bjop.12144 26256821 PMC4849556

[B110] Woodward A. L. Guajardo J. J. (2002). Infants' understanding of the point gesture as an object-directed action. Cogn. Dev. 17, 1061–1084. 10.1016/S0885-2014(02)00074-624795674

[B111] Yu C. Smith L. B. (2016). The social origins of sustained attention in one-year-old human infants. Curr. Biol. 26, 1–6. 10.1016/j.cub.2016.03.026 27133869 PMC5387765

[B112] Zuckerman B. (2009). Promoting early literacy in pediatric practice: twenty years of Reach out and Read. Pediatrics 124, 1660–1665. 10.1542/peds.2009-1207 19917584

